# A morphological investigation of sexual and lateral dimorphism in the developing metanephric kidney

**DOI:** 10.1038/srep15209

**Published:** 2015-10-15

**Authors:** Kieran M. Short, Ian M. Smyth

**Affiliations:** 1 Department of Biochemistry and Molecular Biology, Monash University, Wellington Rd, Clayton, Melbourne, Australia, 3800; 2 Department of Anatomy and Developmental Biology, Monash University, Wellington Rd, Clayton, Melbourne, Australia, 3800

## Abstract

Sexual dimorphism is a prominent feature of renal physiology and as a consequence, it differentially affects predisposition to many adult kidney diseases. Furthermore the left and right kidneys differ in terms of their position, size and involvement in congenital malformations of the urogenital tract. We set out to determine whether differences in the program of branching morphogenesis that establishes the basic architecture of the kidney were apparent with respect to either sex or laterality in mouse embryonic kidneys. This was achieved using a combination of optical projection tomography imaging and computational analysis of many spatial metrics describing the branched ureteric tree. We undertook a comprehensive assessment of twelve aspects of ureteric morphology across developmental time and we found no consistent differences between kidneys of different sexes or laterality. These results suggest that dimorphism is established after birth or at a physiological or cellular level that is not reflected in the morphology of the ureteric tree.

The metanephric kidney of mammals plays a critical role in regulating the excretion of solutes from the blood stream and in the control of complex physiological features such as blood pressure. Increasing evidence suggests that there are considerable gender differences which affect both normal renal function and the capacity of the kidney to respond to damage or disease. Numerous instances of such effects are evident both in human populations (such as acute kidney injury^1^,^2^) and in commonly used mouse models of disease (such as those caused by drugs or ischemia^3^,^4^,^5^,^6^,^7^). Broader measures of renal function such as age related decline in glomerular filtration rate^8^, general predisposition to the development of end stage renal disease^9^ and responses of the renin-angiotensin system^10^ are all influenced by gender. Although there is evidence in some cases that these effects are driven by differences in adult physiology (and the production of sex hormones in particular) it has not yet been established whether these exist as a consequence of differences in renal development.

Asymmetry in the body plan impacts kidney position, with the right kidney more intimately sharing the abdominal cavity with the liver. This is commonly credited as the reason for its more inferior and lateral position. Perhaps as a consequence, the right kidney is slightly smaller than the left^11^. The vascularisation of the kidney is also known to vary based on laterality^12^. While a recent survey of the superficial morphology of the developing human foetal kidney documented the gradual ascension of the organ as development progresses, it found no evidence for sex or laterality effects on renal dimensions, weight (absolute or relative to body weight) or volume^13^. However, evidence for altered lateral development has been postulated, based on the observation that congenital abnormalities of the urogenital tract (CAKUT) are more frequently described on the left rather than the right side^14^. This laterality bias might also be reflected in the observation that left kidney size is reduced in children of low birth weight^15^ and that unilateral multicystic dysplastic kidney is significantly more common in the left versus the right organ^16^. Despite these pronounced differences, their anatomical or physiological bases are currently unclear.

The ureteric bud (UB) sprouts from the nephric duct and invades the metanephric mesenchyme, initiating metanephric kidney development. Through a process of reciprocal interactions between these cell populations, the UB undergoes a program of branching morphogenesis to form an extensive bifurcated tree which will ultimately differentiate to form the collecting duct system of the adult organ. The cellular interactions between the tips of the branching UB and the cap mesenchyme cells surrounding them are important for instructing a proportion of the latter population to undergo mesenchyme to epithelial transition to form the renal vesicles which then remodel and differentiate to eventually form the functional units of the kidney, the nephrons. The association between UB branching and nephron specification is therefore tightly linked. In the mouse, branching morphogenesis occurs most rapidly during the period between the first branch event (at embryonic day (E) 11.5) and E16.5, at which point more than 1300 UB tip niches have been formed, as a result of approximately 13 branching events^17^. This rapid expansion in size and complexity is likely to be instrumental in establishing the normal architecture of the adult organ. The extent to which this critical early period of renal development contributes to (or is responsible for) adult sex or lateral dimorphism has yet to be determined.

## Results and Discussion

**Figure 1:**
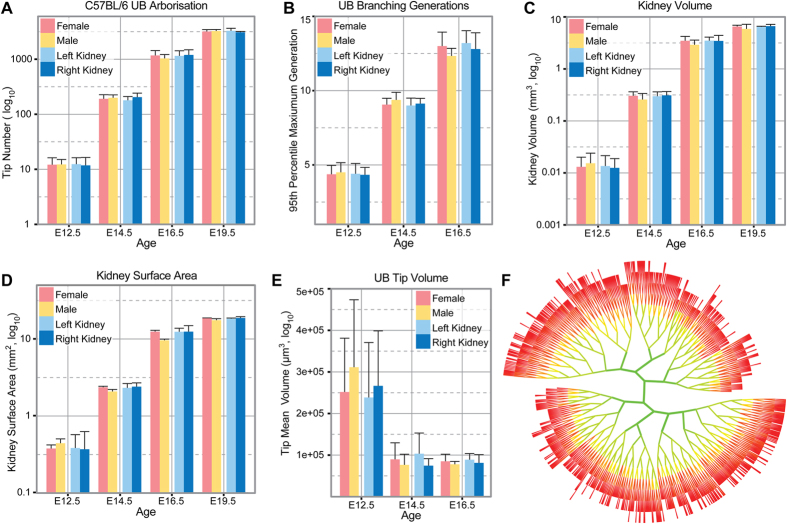
Comparison of UB branching between male/female and left/right kidneys across selected stages of C57Bl6 mouse embryonic development. The number of tips (**A**), the maximum (95^th^ percentile) branching generation (**B**), kidney volume (**C**), kidney surface area (**D**), and tip volumes (**E**) are indistinguishable between sex and laterality. The branching pattern (**F**) is also consistent between all groups across development as noted by the gradient of green (center) to red (tips). No differences were noted in statistical tests, *p* > *0.05* (Supplementary Table 1).

Until recently analysing the contribution of branching morphogenesis to organ formation has been hampered by a lack of methods to image and quantify the branching process. However, we have recently developed tools^18^,^19^ which allow us to comprehensively address whether sexual or lateral dimorphism apparent in the kidney is shaped by differences in the process of ureteric branching morphogenesis. Using the C57BL/6J mouse as our model we have analysed the structure of the branched ureteric tree at embryonic days 12.5 (Theiler stage^20^ 20, limb stage^21^ 8), 14.5 (Theiler stage 22, limb stage 11), 16.5 (Theiler stage 24, limb stage 13) and 19.5 (Theiler stage 26/27, limb stage 14). This time line encompasses most if not all of the branching events that establish kidney structure. Left/right and male/female fetal organs were collected, stained with antibodies to the ureteric epithelium (E12.5 to E16.5) or cap mesenchyme (E19.5, as a measure of tip number^17^), imaged by optical projection tomography and analysed using either Tree Surveyor or Imaris software using our established protocols^19^. We first assessed the branched UB structures by examining metrics describing the UB tips – the site of nephrogenesis and interaction with the cap mesenchyme. Comparisons of gross numbers of tips and branch generations showed that there was no difference in either measure relative to the sex or body side of the embryo across development (Fig. 1A,B; Table 1). It is possible that the number of tips is packed into a smaller area, but we also found that kidney volume and surface area were invariant (Fig. 1C,D). The UB tip volumes are also indistinguishable by sex or laterality across development (Fig. 1E). While tip number might be equal, it may still mask a demonstrable difference in the higher order organ structure – for example branching might be more advanced in some parts of the organ in one area relative to the other. To examine this possibility we compared the cumulative profile of the hierarchical branch pattern of every tip in the organ between sexes and body axes from E12.5 to E16.5. We found that the higher order structure of all kidneys was comparable (indicated by a gradual change in colour from green to red across all clades of the kidney) (Fig. 1F), and reinforces the notion of kidney branching stereotypy^17^. While no overt structural changes were identified, morphological alterations may be reflected in alternative spatial positioning of different tips or branch points in 3D space. To examine this possibility we measured and compared the branch angle and dihedral angles for every bifurcation in the organ. The latter measure quantifies the rotation between successive branch generations. In both cases we assessed local measures which are relative to the initial growth direction of branches from the site of a bifurcation point, and global measures which assess angles from a bifurcation point relative to the bifurcation points (or tips) of its children. These metrics were examined for all branch generations relative to sex and laterality and no statistically significant differences were noted (Supplemental Figure 1A–E).

**Figure 2:**
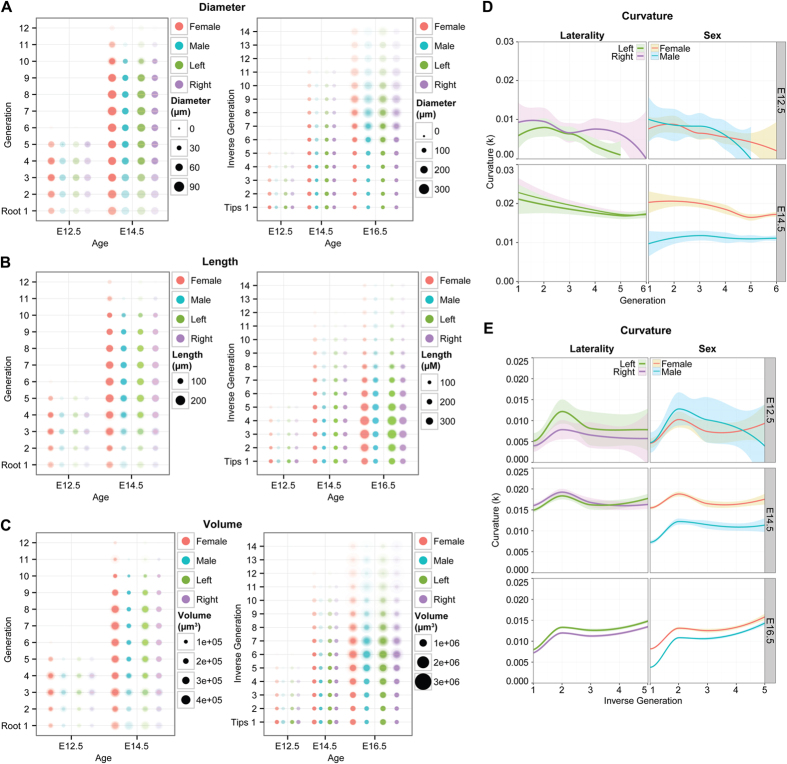
Branch morphology comparisons between sex and laterality of C57Bl6 embryonic mouse kidneys. When assessed by both generation (from the ureter, out) and inverse generation (from the tips, in), the diameter (**A**), length (**B**) and volume (**C**) is consistent, with no significant differences detected (2-factor nested ANOVA). One point of difference is that of branch curvature. When analyzing branches by generation (**D**) or inverse generation (**E**), male and female kidneys differ at E14.5, with female kidney branches having more curvature (Table 2). This is no longer statistically significant by E16.5 (**E**). Note: E16.5 data by generation is not shown due to ambiguity of the first two generations which are subsumed by the pelvis at this developmental stage.

Descriptions of tip/branch number, hierarchy, and location in 3D space might fail to capture more subtle alterations in the structure of the branched UB. To this end we examined the length, volume, average diameter and curvature of the individual segments of the branched tree. In the case of dimensions, we saw no significant differences between embryonic gender or laterality (Fig. 2A–C). Interestingly, while there was no difference when comparing by laterality, there was a quantifiable difference in the curvature of branches between sexes at age E14.5 (Fig. 2D,E), with female branches exhibiting a uniform increase in curvature compared to male organs. While this affect was apparent across branch generation (Fig. 2D) and “inverse” generation (from the tips inward) (Fig. 2E, Table 2) the effect was a transient one, as differences were not mirrored at E12.5 or E16.5.

Taken together this analysis emphasises the robustness of the program of renal branching morphogenesis. The only difference we noted with respect to organ sex or laterality was an increase in the curvature of branches in female embryos at E14.5, but this resolved by E16.5. On this basis we find little supportive evidence for there being either a gender or laterality bias in the branching morphogenesis of the ureteric bud in the C57BL6/J mouse, despite the masculinisation of the organism having commenced by E11.5^22^.

## Methods

### Fetal kidneys

Pure C57BL6/J mice (imported from The Jackson Laboratory in 2012) were pair mated and embryos collected at E12.5, E14.5, E16.5 and E19.5. A total of 12 litters (approximately 3 per time point) were taken. Male/Female animal numbers E12.5 (10/10), E14.5 (5/10), E16.5 (4/5), E19.5 (4/4). Female kidneys were used during laterality analysis to remove any potential sex bias. Kidney numbers used in analyses are available in Table 1 and statistical comparisons of this data in Supplementary Table 1. Visual inspection of the associated gonads was used to determine the sex of the embryo. All animal handling and experimental protocols were approved by the Animal Ethics Committee of Monash University (MARP2012–038) and conformed to the guidelines of the National Health and Medical Research Council of Australia. Mice had *ad libitum* access to standard chow and water with a 12 hour light/dark cycle. Mice were mated overnight. The presence of a vaginal plug the following morning indicated embryonic day 0.5 (E0.5). Embryos were accurately staged by Theiler and limb staging criteria. Animal models were maintained under the auspices of ethics applications to Monash University (Monash Animal Research Platform committee B) and in accordance with the conditions set out by the Australian Bureau of Animal Welfare.

### Organ staining and Optical Projection Tomography (OPT) imaging

The branching UB was stained as per published protocols^17^,^19^. Briefly, organs were fixed in 4% paraformaldehyde in phosphate buffered saline for 10 minutes and stained with mouse pan-cytokeratin (Abcam #115959) primary and anti-mouse Alexa-555 secondary antibodies (Life Technologies #A-31570). As previously published^23^ samples were embedded in agarose, cleared, and scanned using a Bioptonics 3001 OPT scanner (Bioptonics, UK).

### Quantification of 3D UB data

The Tree Surveyor program^18^ was used to profile the three-dimensional structure of the branched ureteric bud. Results were examined comparing metrics for tip number, the number of branch generations, kidney volume and surface area, mean tip volume, branch segment length, volume, diameter and curvature, local and global branch angles and dihedral branch angles. Niche counts and kidney volume/surface area calculations at E19.5 were carried out as previously described^19^ using Imaris (Bitplane, Oxford Instruments).

### Statistical Analyses

T-tests with Welch’s correction for potential unequal variances and unequal sample sizes was used for whole-kidney measurement comparisons. A 2-factor nested ANOVA was used to compare branch metrics between groups. This partitions variation/drift in individual samples inside group variation, before comparison with another like-wise partitioned group. Plotting of data was carried out using R statistics^24^ with the Deducer package^25^, and statistical tests were performed using Microsoft Excel 2010 (with two-factor nested ANOVA formulas^26^, and two-tailed t-tests as standard).

## Additional Information

**How to cite this article**: Short, K. M. and Smyth, I. M. A morphological investigation of sexual and lateral dimorphism in the developing metanephric kidney. *Sci. Rep.* **5**, 15209; doi: 10.1038/srep15209 (2015).

## Supplementary Material

Supplementary Information

## Figures and Tables

**Table 1:** Global comparative kidney metrics for sex and laterality across C57BL/6 development.

**Age**	**Group**	**Generation**	**Tip number**	**Kidney Volume (mm**^**3**^)	**Surface Area (mm**^**2**^)	**Tip Volume (μm**^**3**^)	**n**
E12.5	Female	4.4 ± 0.6	12.6 ± 3.3	0.0142 ± 0.006	0.42 ± 0.21	2.5E5 ± 1.26E5	19
	Male	4.5 ± 0.6	12.5 ± 2.3	0.0167 ± 0.007	0.48 ± 0.19	3.1E5 ± 1.56E5	14
	Left	4.4 ± 0.7	12.8 ± 3.2	0.0148 ± 0.006	0.42 ± 0.18	2.38E5 ± 1.25E5	10
	Right	4.3 ± 0.5	12.3 ± 3.4	0.0134 ± 0.005	0.42 ± 0.23	2.67E5 ± 1.25E5	9
E14.5	Female	9.1 ± 0.4	193.3 ± 30.7	0.308 ± 0.053	2.37 ± 0.27	8.9E4 ± 3.8E4	17
	Male	9.4 ± 0.5	200.5 ± 23.0	0.265 ± 0.058	2.11 ± 0.32	7.6E4 ± 2.4E4	8
	Left	9 ± 0.5	181.1 ± 26.7	0.303 ± 0.056	2.34 ± 0.28	1.0E5 ± 4.7E4	9
	Right	9.1 ± 0.3	207.0 ± 29.2	0.314 ± 0.05	2.42 ± 0.25	7.5E4 ± 1.6E4	8
E16.5	Female	13.0 ± 0.9	1192.8 ± 209.2	3.53 ± 0.590	12.5 ± 1.6	8.5E4 ± 1.6E4	10
	Male	12.3 ± 0.5	1046.8 ± 165.2	2.97 ± 0.640	10.6 ± 1.9	7.8E4 ± 6.5E3	6
	Left	13.2 ± 0.7	1169.2 ± 212.0	3.53 ± 0.479	12.5 ± 1.2	8.9E4 ± 1.3E4	5
	Right	12.8 ± 1.0	1216.4 ± 203.7	3.537 ± 0.683	12.6 ± 1.9	8.1E4 ± 1.8E4	5
E19.5	Female		3196.8 ± 263	6.557 ± 0.39	18.6 ± 0.6		8
	Male		3259.3 ± 211.4	5.999 ± 1.284	17.7 ± 2.4		8
	Left		3300 ± 348.3	6.447 ± 0.178	18.4 ± 0.4		4
	Right		3093.5 ± 107.9	6.583 ± 1.598	18.8 ± 3		4

**Table 2:** E14.5 male/female curvature differences (tested by 2-factor nested ANOVA).

**Branches Analysed**	**Female Group**	**Male Group**	**p-Value**
Generation 1	0.020056	0.009305	0.0460
Generation 2	0.020455	0.011502	0.0438
Generation 3	0.020123	0.012236	0.0349
Generation 4	0.018552	0.011252	0.0064
Generation 5	0.016459	0.010866	0.0074
Inverse Generation 1	0.016625	0.009201	0.0001
Inverse Generation 2	0.018901	0.011752	0.0052
Inverse Generation 3	0.016665	0.011426	0.0184
Inverse Generation 4	0.016977	0.010183	0.0045
Inverse Generation 5	0.017366	0.011899	0.0255
