# Supplementary material for: A morphological investigation of sexual and lateral dimorphism in the developing metanephric kidney
Source: Sci Rep. 2015 Oct 15;5:15209. doi: 10.1038/srep15209 (PMC4606730; doi:10.1038/srep15209)
Supplement: Supplementary Information [file srep15209-s1.pdf]

An investigation of sexual and lateral dimorphism in the developing metanephric kidney

Kieran M. Short and Ian M. Smyth

Supplementary Information

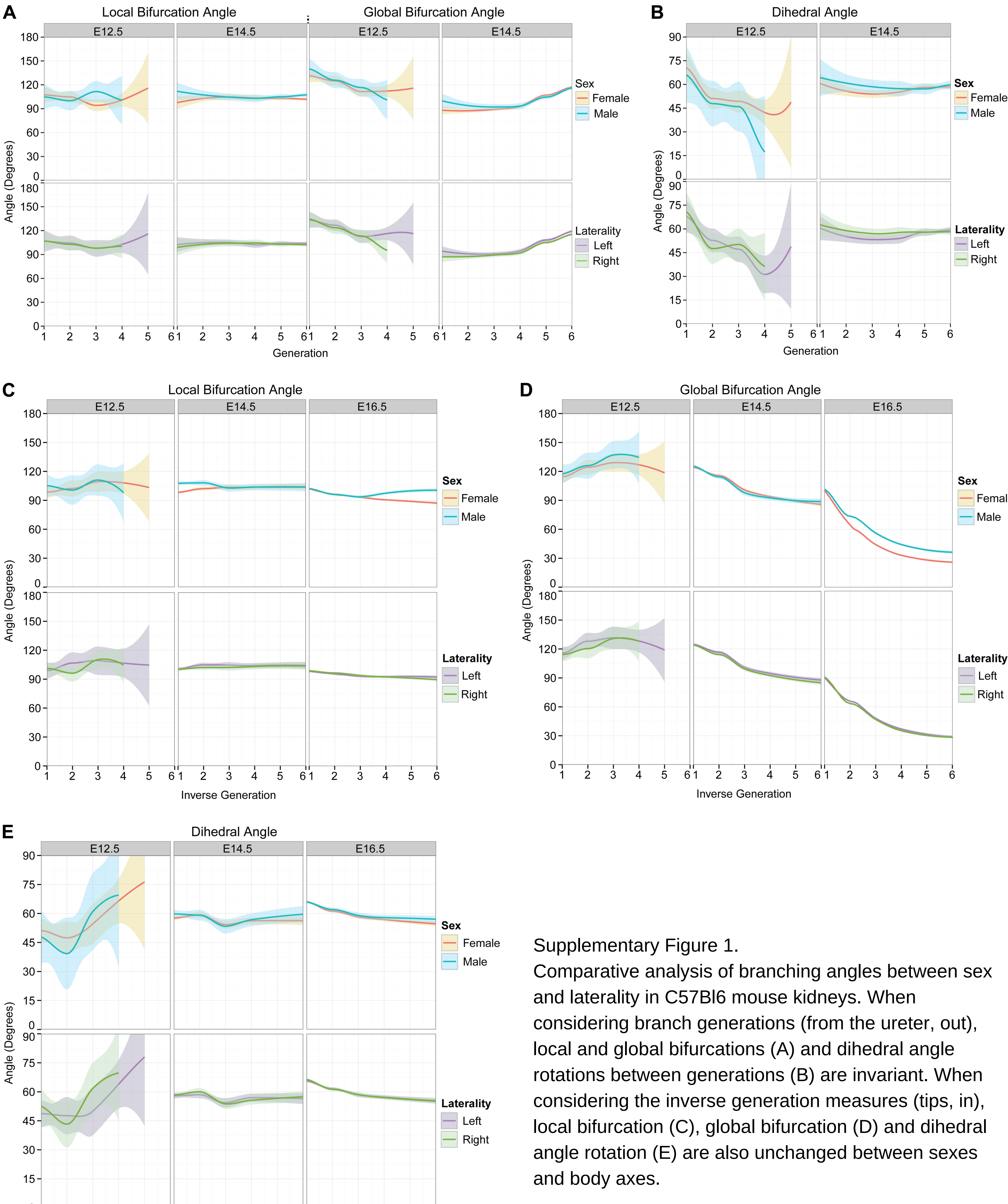

Supplementary Figure 1.
Comparative analysis of branching angles between sex and laterality in C57Bl6 mouse kidneys. When considering branch generations (from the ureter, out), local and global bifurcations (A) and dihedral angle rotations between generations (B) are invariant. When considering the inverse generation measures (tips, in), local bifurcation (C), global bifurcation (D) and dihedral angle rotation (E) are also unchanged between sexes and body axes.

E12.5

Generation		mean	sd	n	p-Value
	Female	4.368421053	0.581335	19	0.544221856
	Male	4.5	0.626783	14	
	Left	4.4	0.663325	10	0.669666091
	Right	4.333333333	0.471405	9	
**Tip Number**		**mean**	**sd**	**n**	**p-Value**
	Female	12.57894737	3.297779	19	0.93589324
	Male	12.5	2.291288	14	
	Left	12.8	3.187475	10	0.797802768
	Right	12.33333333	3.399346	9	
**Kidney Volume**		**mean**	**sd**	**n**	**p-Value**
	Female	14225336.08	5769357	17	0.281331609
	Male	16699806	6588517	14	
	Left	14841434.66	6067976	9	0.998950905
	Right	13455212.86	5272509	8	
**Surface Area**		**mean**	**sd**	**n**	**p-Value**
	Female	418424.4159	205619.2	17	0.348555668
	Male	486508.3929	191258	14	
	Left	415977.7596	182256.8	8	0.999543391
	Right	421919.6391	234954.8	9	
**Tip Volume**		**mean**	**sd**	**n**	**p-Value**
	Female	251780.2872	125844.8	19	0.220460564
	Male	311404.7644	156279	17	
	Left	238493.1393	125405.1	10	0.995212092
	Right	266543.7848	124681.7	9	

E14.5

Generation		mean	sd	n	p-Value
	Female	9.058823529	0.41594517	17	0.13750871
	Male	9.375	0.48412292	8	
	Left	9	0.47140452	9	0.240752564
	Right	9.125	0.33071891	8	
**Tip Number**		**mean**	**sd**	**n**	**p-Value**
	Female	193.2941176	30.7490119	17	0.521590495
	Male	200.5	22.961925	8	
	Left	181.1111111	26.7018287	9	0.514916527
	Right	207	29.1933212	8	
**Kidney Volume**		**mean**	**sd**	**n**	**p-Value**
	Female	308419593	53143681.1	17	0.102455339
	Male	265356744.1	58455595.5	8	
	Left	303205238.2	55527962.2	9	0.999629137
	Right	314285742.1	49676546.2	8	
**Surface Area**		**mean**	**sd**	**n**	**p-Value**
	Female	2377190.032	273157.207	17	0.063870561
	Male	2106932.528	320290.745	8	
	Left	2337951.102	283395.232	9	0.995490325
	Right	2421333.829	254015.319	8	
**Tip Volume**		**mean**	**sd**	**n**	**p-Value**
	Female	89766.40405	38578.7715	17	0.306089267
	Male	76489.50004	24039.3692	8	
	Left	103278.4275	46950.8704	9	0.97248595
	Right	74565.37763	15693.4169	8	

E16.5

Generation		mean	sd	n	p-Value
	Female	13	0.894427	10	0.073244822
	Male	12.33333333	0.471405	6	
	Left	13.2	0.748331	5	0.387907776
	Right	12.8	0.979796	5	
**Tip Number**		**mean**	**sd**	**n**	**p-Value**
	Female	1192.8	209.2199	10	0.148283028
	Male	1046.833333	165.1872	6	
	Left	1169.2	211.9768	5	0.960752245
	Right	1216.4	203.7102	5	
**Kidney Volume**		**mean**	**sd**	**n**	**p-Value**
	Female	3534082569	5.9E+08	10	0.114341485
	Male	2972698340	6.4E+08	6	
	Left	3530965390	4.79E+08	5	0.999995206
	Right	3537199749	6.83E+08	5	
**Surface Area**		**mean**	**sd**	**n**	**p-Value**
	Female	12534809.86	1574789	10	0.069018767
	Male	10634679.09	1896457	6	
	Left	12464662.47	1162483	5	0.999569456
	Right	12604957.25	1897026	5	
**Tip Volume**		**mean**	**sd**	**n**	**p-Value**
	Female	85136.24318	16079.21	10	0.224057626
	Male	77792.35989	6462.741	6	
	Left	89034.75701	13050.68	5	0.984633235
	Right	81237.72936	17786.65	5	

E19.5

Tip Number		mean	sd	n	p-Value
	Female	3196.75	246.015116	8	0.584976992
	Male	3259.25	197.770543	8	
	Left	3300	301.651289	4	0.873908637
	Right	3093.5	93.4465088	4	
**Kidney Volume**		**mean**	**sd**	**n**	**p-Value**
	Female	6557489123	364766573	10	0.366544901
	Male	5998788578	1200676013	5	
	Left	6446575143	154262062	5	0.999651416
	Right	6668403102	466592649	5	
**Surface Area**		**mean**	**sd**	**n**	**p-Value**
	Female	18574022.73	539707.518	10	0.423937558
	Male	17666263.54	2249511.1	5	
	Left	18358932.88	309493.093	5	0.993391187
	Right	18789112.59	627897.4	5	

Supplementary Table1.

Global comparative branching metrics for sex and laterality at E12.5, E14.5, E16.5 and E19.5 during C57BL/6 mouse development with statistical T-test (Welch's T-test) p-values.
